# MicroED beyond the traditional drug space

**DOI:** 10.1063/4.0001032

**Published:** 2025-10-27

**Authors:** Emma Danelius, Guanhong Bu, Tamir Gonen

**Affiliations:** 1 UCR, Riverside, CA, USA; 2 UCLA, Los Angeles, CA, USA

## Abstract

It has been estimated that between 50% and 75% of all proteins in the human proteome are undruggable as therapeutic targets using small molecules residing within Lipinski and Veber’s rules. In the last decades, the discovery of novel therapeutics has undergone a notable transformation driven by the emergence of new chemical modalities which are able to modulate previously “undruggable” targets such as protein surfaces. Due to their flexible structure, these modalities often behave like molecular chameleons, allowing them to alter shape and orientation depending on environmental factors. This chameleonic behavior can enhance their binding affinity, selectivity, and pharmacological activity, however, also makes it challenging to study their structures.

In Microcrystal Electron Diffraction (MicroED) high-resolution structural data are collected from vanishingly small crystals, typically with a thickness in the nanometer range. By circumventing crystal size limitations, MicroED has the potential to enable studies of new and important chemical and biological structures which were previously beyond reach. We have applied MicroED to the study of several systems beyond the traditional drug space as well as their biological targets.

